# Implicit representations of luminance and the temporal structure of moving stimuli in multiple regions of human visual cortex revealed by multivariate pattern classification analysis

**DOI:** 10.1152/jn.00359.2012

**Published:** 2013-05-15

**Authors:** Stephen T. Hammett, Andrew T. Smith, Matthew B. Wall, Jonas Larsson

**Affiliations:** Department of Psychology, Royal Holloway, University of London, Surrey, United Kingdom

**Keywords:** speed, temporal frequency, visual cortex, human, fMRI

## Abstract

The generation of a behaviorally relevant cue to the speed of objects around us is critical to our ability to navigate safely within our environment. However, our perception of speed is often distorted by prevailing conditions. For instance, as luminance is reduced, our perception of the speed of fast-moving patterns can be increased by as much as 30%. To investigate how the cortical representation of speed may vary under such conditions, we have measured the functional MRI blood oxygen level-dependent (BOLD) response of visual cortex to drifting sine gratings at two very different luminances. The average BOLD response in all areas was band-pass with respect to speed (or equivalently, temporal frequency) and thus contained no unambiguous speed information. However, a multivariate classifier was able to predict grating speed successfully in all cortical areas measured. Similarly, we find that a multivariate classifier can predict stimulus luminance. No differences in either the mean BOLD response or the multivariate classifier response with respect to speed were found as luminance changed. However, examination of the spatial distribution of speed preferences in the primary visual cortex revealed that perifoveal locations preferred slower speeds than peripheral locations at low but not high luminance. We conclude that although an explicit representation of perceived speed has yet to be demonstrated in the human brain, multiple visual regions encode both the temporal structure of moving stimuli and luminance implicitly.

the question of how an object's speed is encoded is critical to an understanding of how the visual system guides us in real-world situations. Whereas the cortical pathway responsible for motion processing is relatively well defined, an understanding of the precise mechanisms involved in encoding the speed of a moving image has proven evasive. The earliest (retinal and thalamic) stages of visual processing are mediated by neurones whose responses are spatiotemporally separable (Foster et al. 1985; Tolhurst and Movshon 1975). Such neurones do not provide an unambiguous code for speed. Various models of how the spatiotemporally separable signals generated in the retina are transformed to provide an unambiguous speed code have been proposed (Adelson and Bergen 1985; Hammett et al. 2005, 2007; Langley and Anderson 2007; Perrone 2005; Priebe and Lisberger 2004; Smith and Edgar 1994; Stocker and Simoncelli 2006; Thompson et al. 2006), but there is still no clear picture of exactly where in the pathway speed tuning arises nor how it is achieved. The speed tuning of many middle-temporal visual area (MT) neurones (Perrone and Thiele 2001) and a direct link between their activity and speed perception (Liu and Newsome 2005; Rudolph and Pasternak 1999) raise the possibility that an explicit code for speed may be extracted from early spatially and temporally tuned responses in later cortical areas. However, other studies cast doubt on a straightforward hierarchy of speed encoding across cortical areas and indicate that speed tuning in MT may, at least in part, be inherited from speed-tuned cells in the primary visual cortex (V1) (Priebe et al. 2003, 2006). Priebe et al. (2006) report that ∼25% of V1 and MT neurones have complete spatiotemporal separability, whereas another 25% show clear speed tuning. Thus both spatiotemporally separable and speed-tuned responses may contribute to the neural representation of speed. Indeed, Reisbeck and Gegenfurtner (1999) reported behavioral evidence consistent with such a scheme. In summary, previous models of how the brain encodes speed have been predicated on the assumption that an explicit neural speed code exists. However, electrophysiological evidence for such a code is mixed—some cells as early as V1 are speed tuned, but others are spatiotemporally separable. The same variability is seen in higher cortical areas, such as MT. Thus where such a code is generated is unclear. Moreover, how such a code is generated is equally unknown, and recent electrophysiological evidence (Krekelberg et al. 2006) has failed to find support for any extant model of how speed encoding may be accomplished.

The advent of functional MRI (fMRI) has provided considerable clues as to how the visual system encodes object properties at the neural population level, and a clear coupling of the blood oxygen level-dependent (BOLD) response to fundamental image attributes, such as contrast and motion coherence, has been reported. For instance, Boynton et al. (1999) have shown that the BOLD response increases monotonically as image contrast increases. Given the critical nature of encoding object speed, it is tempting to assume that its cortical representation would be similarly unambiguous. However, the few previous attempts to characterize speed encoding using fMRI have not been entirely consistent and have failed to reveal a straightforward relationship between image speed and BOLD magnitude. Two early studies (Chawla et al. 1998, 1999) used spatially broad-band stimuli (moving dots). They found heterogeneity in the BOLD response, such that whereas later areas (V3 and MT) showed a band-pass response as a function of speed, V1 produced a low-pass response. However, interpretation of their results at high speeds is complicated by the severe undersampling of motion inherent in their stimuli (each dot moved up to eight times its diameter on each frame update), which may explain the reduced response. A subsequent study that used narrow-band stimuli and a more limited speed range (Singh et al. 2000) reported band-pass speed tuning in all cortical areas from V1 to MT. The tuning was both quantitatively and qualitatively similar across the entire visual cortex. A later study of flicker sensitivity (Hagenbeek et al. 2002) also found band-pass tuning in V1. More recently, Lingnau et al. (2009) have reported fMRI adaptation data, suggesting that the BOLD response is more sensitive to speed than temporal frequency, at least in MT and at high contrast. However, their results also provided evidence for temporal tuning across all cortical areas at lower contrast.

In summary, both electrophysiological and imaging studies have provided mixed evidence regarding the locus and nature of speed encoding. There is strong electrophysiological evidence of a direct link between speed perception and activity in MT, but there is also evidence of speed tuning in V1 and spatiotemporally separable responses in MT. Unfortunately, psychophysical studies do not provide any greater illumination given the evidence that the substrates of perceived speed are not unequivocally tuned for either speed or temporal frequency (Thompson et al. 2006). Computational approaches to speed encoding have proven equally unfruitful. Krekelberg et al. (2006) have shown that a Bayesian model, labeled lines, and ratio models (Ascher and Grzywacz 2000; Hammett et al. 2007; Priebe and Lisberger 2004) of speed encoding are all inconsistent with the response characteristics of speed-tuned MT cells. Thus to date, there is no clear account of the evolution of speed encoding nor its locus within the visual pathways.

The absence of a consensus as to how a speed code may be computed renders any parametric study of the BOLD response prone to difficulties of interpretation. One potential way forward is to measure the BOLD response under conditions that are known to affect perceived speed directly. If the BOLD response is coupled to perceived (rather than physical) stimulus speed [see *experiment 2* in Lingnau et al. (2009) for some encouraging, indirect evidence that this may be the case], then modulating perceived speed without changing physical speed should result in a concomitant modulation in BOLD response. The expectation of such a coupling, at least in MT, seems reasonable in light of evidence (Liu and Newsome 2005) that individual neurones in MT play a direct role in speed perception. Thus examining the effect of a stimulus attribute that is known to affect perceived speed on BOLD responses may render a clearer picture of how and where cortical speed encoding occurs.

Recently, Hammett et al. (2007) have shown that perceived speed is modulated by mean luminance, such that low-luminance stimuli appear significantly faster at high speeds. In any cortical region in which perceived speed is represented explicitly, the BOLD response measured under conditions in which both speed and luminance are varied should be affected by both variables, such that it bears a consistent relation to perceived speed. We have therefore measured the BOLD response of various visual cortical areas to drifting sinusoidal gratings at a range of speeds and at two very different luminance levels. In addition, we use multivariate pattern classification analysis (MVPA) to check separately for sensitivity to physical speed and to luminance in each area.

## METHODS

### Experiment 1

#### Subjects.

All seven subjects were undergraduates or postgraduates at Royal Holloway, University of London. All had normal or corrected vision. The experiments were conducted in accordance with the Declaration of Helsinki, approved by a Local Ethics Committee at Royal Holloway, University of London, and written, informed consent was obtained. Standard MRI screening procedures were followed, and volunteers were paid for their participation. Subjects were scanned on two occasions, usually separated by ∼1 wk. Additional scanning runs were also performed on other occasions to define regions of interest (ROIs; see below for details).

#### Data acquisition.

MRI images were acquired with a Siemens 3-Tesla Magnetom Trio scanner with an eight-channel array head coil. Anatomical (T1-weighted) images were obtained at the start of each scanning session [Magnetization-Prepared Rapid Acquisition Gradient Echo, 160 axial slices, in-plane resolution 256 × 256, 1 mm isotropic voxels, repetition time (TR) = 1,830 ms, echo time (TE) = 4.43 ms, flip angle = 11°, bandwidth = 130 Hz/pixel]. This was followed by six scanning runs of functional data acquisition with a gradient echo, echoplanar sequence (TR = 2 s, 28 contiguous axial slices, interleaved acquisition order, 3 mm isotropic voxels, in-plane resolution of 64 × 64 voxels, flip angle = 90°, TE = 30 ms, bandwidth = 1,396 Hz/pixel). Functional scanning runs consisted of 224 volumes and therefore, lasted 7 min, 28 s.

#### Stimuli.

All stimuli were back projected onto a screen mounted in the rear of the scanner bore by a computer-controlled liquid-crystal display projector (Sanyo PLC-XP40L) at a resolution of 1,024 × 768 pixels and a refresh rate of 60 Hz. Participants viewed the stimuli at a distance of 65 cm via a mirror mounted on the head coil, which provided a horizontal viewing angle of ∼30° of visual angle. Stimuli were generated and delivered from Matlab (MathWorks, Natick, MA), using the MGL toolbox (http://www.justingardner.net; http://www.pc.rhul.ac.uk/staff/J.Larsson/dokuwiki/doku.php?id=software).

The stimuli consisted of single, horizontal, sinusoidal gratings, presented in the center of the screen, and ∼20° in diameter. Sine gratings were chosen over broad-band stimuli for comparability with previous work (Hammett et al. 2007). The background and central 1° of the stimulus were set at mean luminance and contained a small, black fixation cross that was present throughout the experiment. The spatial frequency of the grating was one cycle/degree, and the grating drifted upward throughout each trial. Four different speeds were used for the drifting grating, which defined the four conditions: 2°/s, 4°/s, 8°/s, and 13.3°/s. Because the stimuli were sine gratings, and spatial frequency was invariant, the gratings can equivalently be regarded as drifting at 1, 4, 8, and 13.3 Hz. The luminance of the stimulus was controlled by two cross-polarizing filters mounted between the projector and the back-projection screen. By rotating one filter with respect to the other, a continuous modulation of the output of the projector could be achieved, which enabled very precise adjustment with no change in contrast or gamma. Average luminance levels across the stimulus area were measured using a photometer to set the levels used in the experimental sessions. The high-luminance condition's space-average luminance was 30 cd/m^2^, and the low-luminance condition was set at 1.5 cd/m^2^, similar to the luminance levels found by Hammett et al. (2007) to modulate perceived speed.

#### Procedure.

The main experiment was a standard event-related design for fMRI. Each trial lasted 3 s and consisted of a continuous presentation of the drifting grating. The duration of intertrial intervals (ITIs) was drawn randomly from a Poisson distribution (Hagberg et al. 2001) with a range of 2–10 s and a mean of 5.5 s. There were 10 trials of each speed condition (40 in total) in a single scanning run. Each scanning run also included a 10-s buffer period at the beginning and end of the sequence. Each participant completed six scanning runs, lasting ∼7½ min in each scanning session. The six sequences of trial orders and ITIs were the same for every participant, but each participant was exposed to them in a different, random order. The scanning runs alternated between high and low luminance. For three of the participants, the first scanning run was under high-luminance conditions; for the remaining four, the first scanning session was under low-luminance conditions. A tightly fitting black-out screen was placed over the window between the scanner room and the control room, and all other sources of light in the scanner room were either switched off or blocked so that no stray light could influence the luminance levels used in the experiment.

Retinotopic visual areas V1–V3, V3A/B, V7, and human V4 (Larsson and Heeger 2006) were identified by a standard retinotopic mapping procedure (Sereno et al. 1995), using a counter-phasing checkerboard “wedge” stimulus (a 24° sector) of radius 12°. MT/medial superior temporal area (MT/MST, henceforth referred to as MT+) was identified on cortical flatmaps as the set of voxels that showed a significant response to the moving grating stimuli (defined arbitrarily as having a coefficient of determination R^2^ > 0.25 in the event-related model fit described below), using retinotopic criteria (Huk et al. 2002) to distinguish MT+ from nearby motion-responsive areas, such as V1 and V3A. Contrast reversed at a rate of 8 Hz. The wedge rotated clockwise at a rate of 64 s/cycle, and eight cycles were presented.

#### Data analysis.

Data were analyzed using bespoke software, implemented in Matlab (MathWorks) and C/C++. Functional data were preprocessed to correct for head motion using the MCFLIRT tool in FSL (Jenkinson et al. 2002) and filtered with a temporal high-pass filter with a cutoff of 0.025 Hz. No spatial smoothing was performed on the functional data. Functional images from both scanning sessions were coregistered to a high-quality anatomical image (MDEFT) (Deichmann et al. 2004)—hereafter referred to as the reference anatomy—of the same participant, acquired in a separate scanning session. The reference anatomy was also used to extract cortical surface representations using the public domain software SurfRelax (Larsson 2001).

Retinotopic data were analyzed by fitting a sinusoid with a frequency corresponding to the period of the rotating wedge stimulus to the time course for each voxel (high-pass filtered with a cutoff of 0.025 Hz and converted to percent signal change). For each voxel, this yielded a correlation (technically, coherence), a phase, and an amplitude. The phase of the fitted response corresponded to the visual-field (polar angle) location of each voxel. The phases of voxels with a coherence >0.25 were visualized on computationally flattened cortical surfaces, and boundaries between visual areas were identified as reversals of the direction of phase change across the cortical surface (Sereno et al. 1995). ROIs were drawn by eye based on these boundaries.

#### Event-related analysis.

fMRI data were analyzed by fitting a model of the mean fMRI response to the speed stimuli in two steps. First, the trial-triggered average fMRI response to each speed within a session was estimated using deconvolution (linear regression) (Burock and Dale 2000), yielding an individual estimate of the hemodynamic response function (HRF) for each subject and speed. Estimation of the HRF for each subject in this way, rather than using a model of the HRF, allowed us to better capture the intersubject variability in hemodynamic responses and obviated the need for making a priori assumptions about the specific shape of the response. Second, the average fMRI response (or HRF) was used to estimate the amplitude of the responses evoked by each individual stimulus presentation, using a general linear model (GLM) analysis. Although the shape and delay of the trial-triggered responses (HRFs) differed between subjects, it did not differ across speeds, ROIs, or luminance levels, and hence, HRFs were averaged across speed, luminance, and ROIs and normalized to unit area individually for each subject. This analysis was carried out both for each voxel separately (to identify MT+, as described above) and for each ROI (by first averaging the time courses of all voxels within the ROI). In the GLM analysis, each trial was modeled by a separate column in the design matrix, each column containing a copy of the trial-triggered average fMRI response averaged across speeds and ROIs, time shifted to align it with the onset of the trial. The regression coefficients (obtained by standard multiple-regression methods) then corresponded to the fMRI response amplitudes for each trial. The model was fitted separately to the fMRI time series (detrended by high-pass filtering and normalized to zero mean and percent signal change) for each ROI. From these amplitudes, we computed the mean responses (averaged across subjects and trials) for each speed and luminance condition. We chose to use this method to estimate average response amplitudes rather than using the peak amplitudes from the deconvolution analysis, as it allowed us to compare directly the results of the ROI-based analysis with those of the multivariate analysis described below.

#### Multivariate pattern classification analysis.

In a complementary analysis, MVPA, based on sparse regression techniques, was applied to investigate whether the multivariate fMRI response across all voxels in each area could accurately predict the speed of the stimulus for individual trials. We used the least angle regression with least absolute shrinkage and selection operator (LARS-LASSO) algorithm for sparse regression (Efron et al. 2004) to select automatically the voxels that contributed most to the decoding accuracy and overcome the problem of fitting a regression model with more covariates than observations. For every voxel within each ROI, we estimated the response amplitudes for individual speed trials using the same regression model described above. This yielded an [m × n] matrix of fMRI response amplitudes for each ROI, where m (number of rows, 240) is the number of trials (across all speeds), and n is the number of voxels in each ROI.

For each ROI and separately, for each subject and luminance, the fMRI response amplitudes (normalized to unit magnitude within each ROI) were used as covariates to predict the speed on each trial within each session by LARS with the LASSO algorithm. A leave-one-out procedure was used to validate the regression as follows. Data from five out of the six scans in each session were used to fit the regression model, using the voxel-response amplitudes as covariates and the speed on each trial as the dependent variable. Because comparisons of classification performance across ROIs are complicated by differences in size (i.e., number of voxels), response strength, and intrinsic response properties of the ROIs, we tried several criteria for selecting voxels to ensure that our results were not due to the particular selection procedure used. Although the LARS-LASSO algorithm automatically selects the most informative voxels for classification, the algorithm can be constrained to use fewer voxels than the maximum possible (which is given by the number of trials). We explored the effect of varying the number of voxels on classification performance and observed no qualitative difference in the results. However, in all ROIs, we found that classification accuracy peaked at a relatively small number of voxels (25), suggesting that inclusion of additional voxels provided largely redundant information and thus resulted in overfitting. In the following, we show the results obtained using 25 voxels within each ROI but emphasize that the results would have been qualitatively similar with a different number of voxels for all ROIs. The estimated regression coefficients were then applied to the data from the remaining scan to generate predicted speeds for each trial in that scan. This procedure was repeated for each of the six scans. The predicted speeds for all trials in a session were concatenated and the Pearson correlation computed between predicted and actual speeds on each trial to assess performance of the classification procedure. For each actual speed, the predicted speed across all trials was averaged within each subject and luminance. The overall accuracy of the classification was then assessed by plotting the mean predicted speed for each subject and luminance against the actual speed and by computing the Pearson correlation between actual and predicted speeds across subjects. For areas in which the multivariate fMRI response across voxels encodes information about speed, this analysis would predict a monotonic increase in predicted speed with actual speed.

As a control, the MVPA analysis was also carried out on the same data but with the speed labels permuted randomly on the training set, only to estimate chance-level performance of the classification algorithm. The classification procedure described above was repeated 1,000 times for each ROI. On each randomization iteration, the speed labels for all trials were permuted randomly and the classification analysis rerun. For each actual speed and luminance, the chance level-predicted speed was computed as the average across randomization iterations. With the assumption of no bias in the randomization or the classification, this analysis would be expected to predict the same speed (equal to the average of presented speeds) for each actual speed presented. If the multivariate response across voxels in each area carried no systematic information about presented speed, then the result of the original classification (using the correct speed labels) would have been expected to give the same result as the random classification, i.e., a flat-speed prediction equal to the average of the speeds presented; conversely, a systematic deviation from a flat prediction would indicate that the multivariate response across voxels encoded information about presented speeds.

In a second control analysis, we tested directly whether MVPA provided more information about stimulus speed than the original univariate (ROI) analysis. For this analysis, we collapsed the (non-normalized) response amplitude data used for the MVPA analysis across all voxels within each ROI and then subjected the resulting univariate response to the same regression analysis used for the multivariate response. For univariate data, this reduces to a simple regression analysis with a single covariate, but the results had the same dimensions and format as the multivariate analysis, allowing a direct comparison to be made.

### Experiment 2

#### Subjects.

Six subjects (age range 19–49, two men) took part in *experiment 2*—one of whom had also been a subject in *experiment 1*.

#### Stimuli and procedure.

Motion stimuli were identical to those used in *experiment 1*, except that only three speeds were used (3°/s, 6°/s, and 10°/s). The range of speeds was chosen to be within the range of speeds in *experiment 1*. For some of the participants, slightly different procedures and stimuli were used to identify retinotopic visual areas (Larsson and Heeger 2006). For these participants, stimuli consisted of radially moving checkerboards presented within a wedge 22.5° wide and extending from the center of gaze to 13° eccentricity, rotating clockwise or counter-clockwise, step-wise by one wedge width every 1.5 s (synchronized with the scanner TR). Each stimulus cycle lasted 24 s, and six cycles were presented. Data for clockwise and counter-clockwise runs were averaged, as described previously (Larsson and Heeger 2006). For each participant, the experiment was divided into two back-to-back blocks: one for all of the low-luminance conditions and the other for the high-luminance conditions. The order of the low- and high-luminance blocks was counterbalanced across participants. Hence, for three subjects, the high-luminance conditions were run first, followed by the low-luminance conditions, and for the remaining four subjects, blocks were run in the reverse order. Each block was preceded by a short period of adaptation to the appropriate luminance level.

#### Data analysis.

Data were analyzed using the same procedures as in *experiment 1*. For the MVPA analysis, the LARS-LASSO classification algorithm was trained on data from high- or low-luminance runs separately and then tested on data from high- or low-luminance runs, resulting in four estimates of classification performance for each speed (train high, test high; train high, test low; train low, test low; train low, test high). In addition, we trained the algorithm on data from high- and low-luminance runs combined and tested on data for each luminance separately, yielding two additional estimates of classification performance (train all, test high or low).

In a complementary analysis, we used the same multivariate analysis techniques to test whether stimulus luminance, rather than speed, could be decoded from the multivariate response. The analysis was identical to that for speed, except that the decoder was trained to distinguish luminance levels (coded as dummy variables, with zero indicating low luminance and one indicating high luminance), ignoring speed.

#### Estimating the spatial distribution of tuning for speed and luminance.

To test whether responses to speed and/or luminance varied as a function of eccentricity in early visual areas, we mapped the distribution of voxel-wise fMRI speed preferences at high and low luminance across the cortical surface in areas V1, V2, and V3 as follows. First, with the use of the voxel-wise response amplitudes used in the MVPA analysis, we computed a t-statistic for each speed and luminance for each voxel within these areas. Separately for each luminance, we then assigned each voxel a label, corresponding to the speed that yielded the maximal t-statistic (i.e., the most significant response). For each area, we subdivided the region corresponding to the visual stimulus (identified by voxels, showing a significant, increased response to the speed stimuli, defined as the voxels with a R^2^ > 0.25) into 10 bins along the cortical surface in the eccentricity dimension, extending from the representation of the inner (perifoveal) boundary of the stimulus to the outer (peripheral) boundary of the stimulus. The bins had equal width in terms of cortical distance. Data for the two most peripheral bins were excluded, as only a small proportion (<10%) of voxels in these bins met the criteria for inclusion (R^2^ > 0.25 and positive stimulus-evoked BOLD responses). Speed preferences were averaged across voxels within each bin and then averaged across subjects to yield a plot of average speed preference as a function of cortical distance from fovea to periphery for each luminance level. A resampling test was used to assess whether the difference in slopes between high- and low-luminance conditions was significant. For this test, a regression line was fit to the bin-wise difference between high- and low-luminance condition data to yield a regression coefficient *b* and a correlation coefficient *r*. Speed preference data for each subject, hemisphere, and luminance condition were then assigned randomly to one of two equal-sized sets. A regression line was then fit to the bin-wise difference between the two sets in the same way as for the real data. This procedure was repeated 10,000 times to provide an estimate of the probability of obtaining a difference in slopes between the two sets by chance. The significance of the actual difference in slopes was estimated by computing the proportion of resampling iterations where the difference in slopes *b* and the regression coefficient *r* was greater than or equal to the observed ones.

## RESULTS

In all our results, we use the term “speed” to describe the stimuli. However, it should be borne in mind that in terms of tuning of neural mechanisms, our stimuli do not differentiate speed from temporal frequency. Thus our speed-tuning functions can equivalently be regarded as temporal frequency-tuning profiles, and speed decoding can equivalently be regarded as temporal frequency decoding.

## Experiment 1

**Fig. 1.**
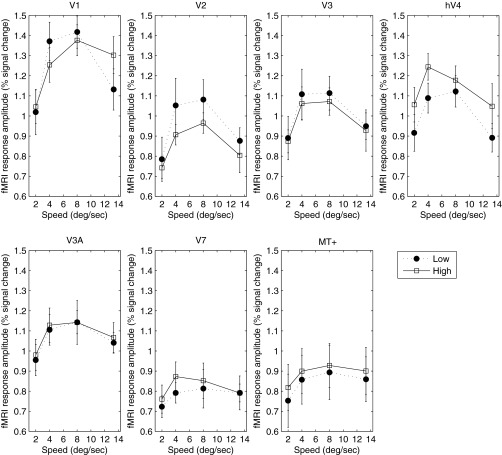
Blood oxygen level-dependent (BOLD) response as a function of speed for low (closed symbols) and high (open symbols) luminance in *experiment 1*. Cortical area is indicated above each panel. Error bars represent ±1 SE. V1, primary visual cortex; hV4, human V4; MT+, middle-temporal visual/medial superior temporal area; fMRI, functional MRI.

Fig. 1 plots the tuning of the BOLD response as a function of speed for all areas measured. All areas show a clear band-pass tuning of the BOLD response at both low and high luminance. The functions in Fig. 1 assume that subjects were able to maintain good fixation. It is possible that some degree of following eye movement may have occurred, despite the use of an unstimulated region immediately around the fixation spot. If so, this would tend to reduce retinal speed, which could distort the results. However, the results are both qualitatively and quantitatively consistent with those of Singh et al. (2000). Importantly, we observed no evidence of a consistent difference in speed response between the high- and low-luminance conditions.

**Fig. 2.**
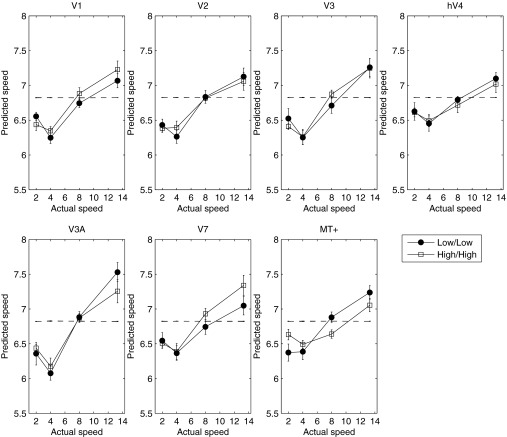
Predicted speed of the classifier as a function of stimulus speed is plotted for high (open symbols) and low (closed symbols) luminance in *experiment 1*. Broken lines represent classifier performance for random assignment of speeds. Cortical area is indicated above each panel. Error bars represent ±1 SE.

**Fig. 3.**
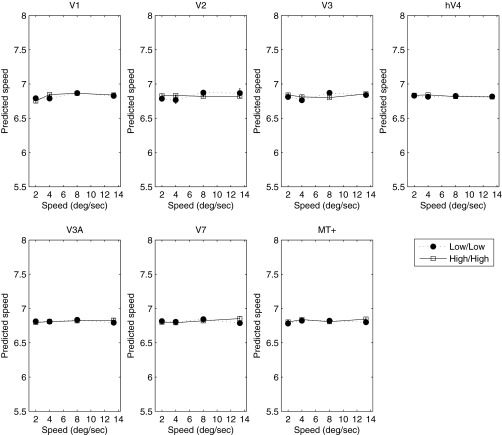
Results of univariate classification analysis. In each area, collapsing data across voxels reduce classifier performance to chance levels. Cortical area is indicated above each panel. Error bars represent ±1 SE.

Given the absence of any reliable change in the BOLD response as a function of luminance, we wondered whether an explicit code for speed (or temporal frequency) might be found in a subpopulation response. To investigate this, we conducted a MVPA classification. Fig. 2 plots the results of the MVPA analysis across all areas and at both luminance levels. The MVPA classifier could not distinguish the two slowest speeds measured but was able to distinguish them from higher speeds. All higher speeds (>4°/s) were predicted unambiguously in all cortical areas and at both luminance levels. At 2°/s, the classifier was unable to discriminate speed, and the predicted speed approached the value of the mean speed of those presented. Excluding the slowest speed, in all areas the classifier's predicted speed rose monotonically with stimulus speed. In a control condition, we also trained the MVPA classifier with random assignment of stimulus speeds. As expected, under these conditions, the classifier was unable to predict any speed, with all stimulus speeds classified around the mean of the four stimulus speeds (shown in Fig. 2). Furthermore, to confirm that the performance of the classifier relied on information contained only in the multivariate response, we ran the classification analysis on the (non-normalized) multivariate data collapsed across voxels, reducing the analysis to a simple regression of speed against the average response across voxels. As expected, given the inherent ambiguity in the band-pass univariate response profile (Fig. 1), the classifier failed to distinguish the different speeds from the average response (Fig. 3). This result demonstrates that although the univariate response does not encode speed unambiguously, information about stimulus speed >2°/s is reliably encoded in the multivariate response.

### Experiment 2

Whereas the classifier could discriminate most speeds, its predictions did not differ between low and high luminance. We reasoned that any shift in the speed code with luminance should be revealed by training the classifier with low luminance and subsequently, predicting the speed of the high-luminance responses (or vice versa). If the classifier-predicted speed corresponded to perceived speed, we might expect a classifier trained at high luminance and tested on high luminance to show systematic deviations in predicted speed at low luminance that matched those observed perceptually. However, it was not tenable to examine the effect of such cross-classification, since each luminance condition was measured in separate scans; thus any differences in performance between within- and across-luminance classification could be explained by differences in the exact voxel positions between scanning sessions. To investigate whether the speed code would reveal luminance dependency under cross-classification, we repeated our measurement for both luminances within the same scanning session, reducing the number of speeds to three to retain an acceptable total scan time.

**Fig. 4.**
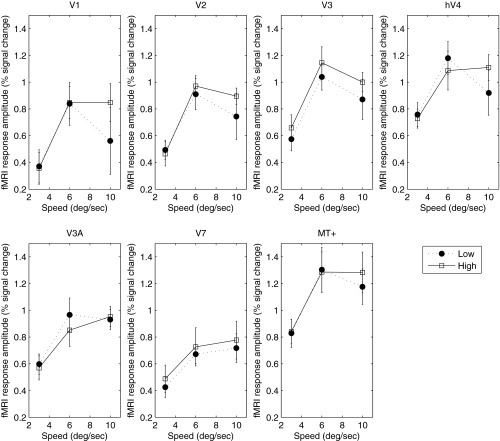
Mean BOLD response as a function of speed for low (closed symbols) and high (open symbols) luminance in *experiment 2*. Cortical area is indicated above each panel. Error bars represent ±1 SE.

The speed-tuning functions of the BOLD response were very similar to and consistent with those obtained in *experiment 1* (Fig. 4). With the exception of V3A and V7, all areas showed band-pass tuning for speed. Since the maximum speed in *experiment 2* (10°/s) was lower than that in *experiment 1* (13.3°/s), the monotonic increase in BOLD response with speed in V3A and V7 is consistent with band-pass tuning, with a peak response at or beyond 10°/s, as suggested by the results of *experiment 1* (Fig. 1).

**Fig. 5.**
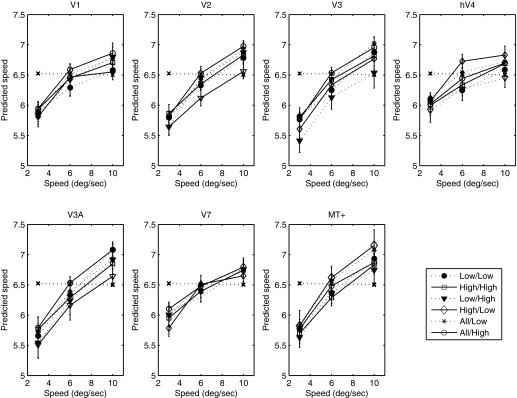
Predicted speed of the classifier as a function of stimulus speed is plotted for high (open symbols) and low (closed symbols) luminance and for low-high and high-low training regimes in *experiment 2*. Broken lines represent classifier performance for random assignment of speeds. Cortical area is indicated above each panel. Error bars represent ±1 SE.

The results of the MVPA analysis for *experiment 2* are plotted in Fig. 5. As in *experiment 1*, in all areas, the classifier's predicted speeds rose monotonically with physical speed. However, contrary to our hypothesis, there was no discernible difference in the predicted speeds between the within- and across-luminance classification conditions.

**Fig. 6.**
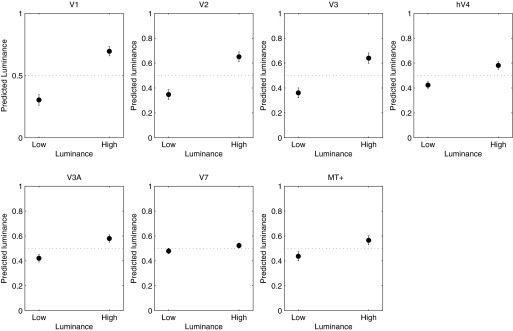
Predicted luminance as a function of actual luminance. Cortical area is indicated above each panel. Error bars represent ±1 SE.

The lack of a consistent effect of luminance on response amplitude or decoding performance could indicate that luminance has little effect on visually evoked cortical responses, as suggested by results showing little or no modulation of fMRI BOLD response in V1 over a wide range of luminance levels (Hadjikhani and Tootell 2000). Alternatively, these results could indicate that luminance is encoded in a cortical BOLD response but in a manner that does not systematically influence response magnitudes. To distinguish between these hypotheses, we used the MVPA analysis to decode luminance instead of speed, training the classifier on data across low- and high-luminance runs and ignoring speed. The results of this analysis are plotted in Fig. 6. With the possible exception of V7, the classifier decoded stimulus luminance successfully in every visual area examined, with early visual areas (V1–V3) showing the most accurate decoding performance. These results show that luminance is indeed encoded in the multivariate BOLD response, although these effects appear not to bias speed responses or speed decoding.

#### Spatial Distribution of Speed and Luminance Tuning

**Fig. 7.**
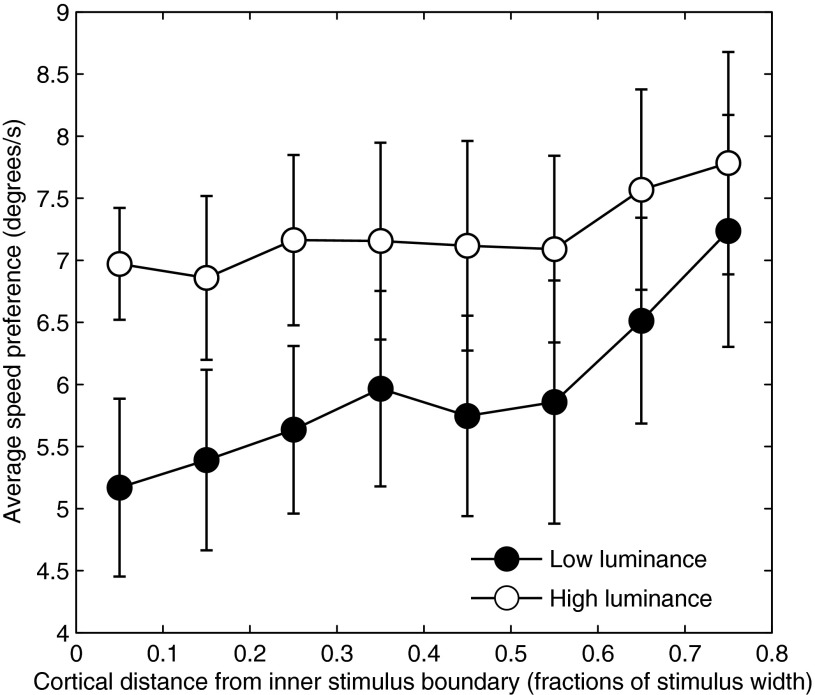
Preferred speed as a function of cortical distance from stimulus boundary for high (open symbols) and low (closed symbols) luminance. Error bars represent ±1 SE.

The observation that both speed and luminance could be decoded accurately from the multivariate BOLD response but not in the overall response averaged across all voxels indicated that voxels differed in their motion and luminance preferences. We wondered whether such differences would show a large-scale spatial distribution, specifically with regards to eccentricity. Magnocellular and parvocellular neurones differ in temporal frequency tuning, luminance, and contrast response (Kaplan et al. 1990; Purpura et al. 1988) and are heterogeneously distributed across visual eccentricity (Dacey 1994); thus the differences in speed/frequency and/or luminance preference could reflect such differences. To investigate this possibility, we plotted speed preference across the cortical surface along the eccentricity dimension in V1 for high- and low-luminance conditions averaged across subjects (Fig. 7). For the low-luminance condition, a clear pattern was observed in that perifoveal locations preferred slower speeds than peripheral locations. The high-luminance condition also shows an effect of eccentricity, but the slope of the function is reduced, such that the two functions are well separated in the parafovea but converge in the periphery. Although we found no overall effect of luminance at any speed in *experiment 1* (Fig. 1), the finding that the preferred speed of parafoveal voxels varies with luminance suggests that localized effects of luminance may exist (but see discussion for consideration of the possible effects of the measurement method).

The difference in slope between high and low luminance was statistically significant (resampling test, *P* < 0.05). A similar but weaker and nonsignificant pattern was observed in V2 and V3 (data not shown). Hence, at least for V1, there appears to be large-scale variation in speed preference with eccentricity that differs between high and low luminance, suggesting that selectivity for speed and luminance interacts in a systematic manner in this area.

## DISCUSSION

Our results demonstrate that at least for narrow-band stimuli, the BOLD response to speed is band-pass, peaking between 4°/s and 10°/s in all cortical areas measured and at both luminances measured. Comparison of our results with those obtained for high luminance by Singh et al. (2000) indicates good quantitative agreement over the speed range tested in all areas. Thus the global population response does not render an unambiguous speed signal. This is perhaps unsurprising, given the large body of electrophysiological evidence that both speed and spatiotemporally separable responses are found in the visual cortex. The BOLD response to a drifting grating in a given cortical region must be assumed to reflect the sum of activities of all mechanisms that exist in that region. Thus the band-pass tuning of the average BOLD response reflects the composite activity of speed- and temporal frequency-tuned neurones. There is considerable psychophysical evidence for the existence of two (or possibly three) nondirectional, temporally tuned mechanisms (Hammett and Smith 1992; Hess and Snowden 1992; Kulikowski and Tolhurst 1973), whose overall response envelope is band-pass, peaking approximately 6–8 Hz. Whereas the precise relation of these mechanisms to underlying neural physiology is far from clear, they do share temporal-tuning characteristics that are broadly similar to the temporal tuning of magno- and parvocellular cells, the outputs of which presumably form the substrate for speed-tuned neurones (De Valois et al. 2000). Regardless of its precise neural substrate, our results are consistent with previous studies (Singh et al. 2000), indicating that the average BOLD signal contains no unambiguous information regarding speed. It should again be noted that because we, like previous studies, have used only one spatial frequency, we cannot disambiguate speed and temporal frequency, and thus our result may reflect coding of flicker rather than speed per se.

However, the results of the MVPA classification indicate that although speed information is not encoded unambiguously in the global response, the multivariate response across voxels within each visual area does contain information about speed. Whereas the MVPA classifier could not distinguish the two slowest speeds measured in *experiment 1*, it could distinguish them from higher speeds, each of which was also predicted unambiguously. Thus whereas the average BOLD response conveys no unambiguous speed information, the multivariate response in all cortical areas measured yields clear information about the speed of the stimulus over most of the range tested. However, we found no difference in either the mean BOLD response or the multivariate response across luminance and no difference in the classifier-predicted speed applied within or across luminance conditions. This is surprising, given the known variation in perceived speed with luminance (Hammett et al. 2007; Vaziri-Pashkam and Cavanagh 2008) and the known changes in response of retinal ganglion cells with luminance (Purpura et al. 1988). We know of few studies that have measured the fMRI BOLD response directly as a function of luminance, and it may be that early (retinal) luminance gain control effectively renders the BOLD response relatively immune to luminance modulation. Indeed, Hadjikhani and Tootell (2000) have shown that a million-fold reduction in luminance yielded little change in activity in areas from V1 to V4. Moreover, Cornelissen et al. (2006) report that neither luminance nor perceived luminance modulations are accompanied by commensurate changes in the BOLD signal in V1 and V2, and Leonards et al. (2005) report a cortical region tuned for luminosity perception that does not increase its activity with increasing luminance.

Whereas we found no systematic change in overall BOLD response with luminance, consistent with previous studies, MVPA analysis could decode luminance successfully in almost every visual area, indicating that just as for speed, information about stimulus luminance is encoded in the multivariate response across voxels. The lack of effect of luminance on speed decoding would thus suggest that speed and luminance are encoded independently in visual cortex. Such a conclusion is, however, at odds with the effect of luminance on variations in speed preference as a function of eccentricity that we observed in V1, which indicated an interaction between speed and luminance. How can these two seemingly contradictory observations be reconciled? It is likely that the difference in results is, at least in part, due to differences in analysis methods. The variation in speed preference with eccentricity was computed by assigning to each voxel the speed label corresponding to the speed that evoked the most significant (rather than strongest) response. This is essentially a “winner-takes-all” algorithm, which being inherently nonlinear, can potentially exaggerate differences in responses to different speeds, even if the underlying response differences are small. Moreover, the computation of speed preferences was performed only on voxels showing a positive BOLD response (increase) to the speed stimuli, whereas the classification analysis was performed on all voxels showing a significant stimulus-evoked response, whether positive (increase) or negative (decrease). Indeed, the observation that speed classification was equally or more accurate in V2 and V3 than in V1, even though there was much less evidence of a systematic eccentricity effect on speed preference in these areas than in V1, suggests that the classifier was not driven primarily by eccentricity-specific variations in speed preference. Similarly, for the high-luminance condition, speed preference did not vary with eccentricity in V1, yet speed classification performance was no worse for the high- than the low-luminance conditions. Together, this suggests that the effect of eccentricity on speed preference did not bias classifier performance, even if the classifier may, in part, have relied on information contained in these large-scale biases. Although there is controversy about the spatial scale of BOLD responses underlying MVPA (Freeman et al. 2011; Kamitani and Sawahat 2010), it is likely that information in the BOLD response may be found at several spatial scales, all of which may drive classification performance (Chaimow et al. 2011). The fact that the speed classification analysis did not show a sensitivity to luminance could thus reflect the existence of smaller-scale speed biases in the multivariate response, which might have been invariant to luminance differences. Whereas the classification results indicate that information about speed and luminance is encoded independently at the coarse scale of the multivariate BOLD response, these results do not rule out that the two stimulus features interact at the single neurone level, which could potentially account for the effect of luminance on perceived speed.

In summary, our results are broadly consistent with a scheme, whereby the average BOLD response reflects the envelope of neural responses, many of which may not be speed tuned but whose multivariate response contains unambiguous speed (or temporal frequency) information in all visual areas measured. We propose that the most parsimonious account of our results is that speed encoding is only represented implicitly in visual cortex and is estimated from the multivariate population response. How could such an implicit code for speed be realized? One possibility is that rather than a speed code being extracted upon the basis of the tuning of speed-sensitive cells, either the responses of discrete, neural subpopulations or the multivariate response of entire regions are used to render a speed-related signal. Our results do not speak directly to any specific model of speed perception, but they do indicate that speed or temporal frequency tuning may occur as early as V1 and that any model must reflect either the response properties of subpopulations of neurones within motion-sensitive areas or the multivariate population response of those areas.

## GRANTS

Support for this work was provided by Wellcome Trust Grant GR065624 to S. T. Hammett.

## DISCLOSURES

No conflicts of interest, financial or otherwise, are declared by the authors.

## AUTHOR CONTRIBUTIONS

Author contributions: S.T.H., A.T.S., M.B.W., and J.L. conception and design of research; S.T.H., M.B.W., and J.L. performed experiments; M.B.W. and J.L. analyzed data; S.T.H., A.T.S., M.B.W., and J.L. interpreted results of experiments; S.T.H. and J.L. prepared figures; S.T.H. drafted manuscript; S.T.H., A.T.S., M.B.W., and J.L. edited and revised manuscript; S.T.H. approved final version of manuscript.
